# CD4^+^ T-Cell Senescence in Neurodegenerative Disease: Pathogenesis and Potential Therapeutic Targets

**DOI:** 10.3390/cells13090749

**Published:** 2024-04-25

**Authors:** Yan Gao, Yaoping Lu, Xiaojing Liang, Mengwei Zhao, Xinyue Yu, Haiying Fu, Wei Yang

**Affiliations:** Department of Immunology, College of Basic Medical Sciences, Jilin University, Changchun 130021, China; yangao22@mails.jlu.edu.cn (Y.G.); luyp21@mails.jlu.edu.cn (Y.L.); liangxj22@mails.jlu.edu.cn (X.L.); zhaomw23@mails.jlu.edu.cn (M.Z.); yuxy2819@mails.jlu.edu.cn (X.Y.); fuhy@jlu.edu.cn (H.F.)

**Keywords:** immunosenescence, CD4^+^ T-cells, neurodegenerative disease, therapy

## Abstract

With the increasing proportion of the aging population, neurodegenerative diseases have become one of the major health issues in society. Neurodegenerative diseases (NDs), including multiple sclerosis (MS), Alzheimer’s disease (AD), Parkinson’s disease (PD), and amyotrophic lateral sclerosis (ALS), are characterized by progressive neurodegeneration associated with aging, leading to a gradual decline in cognitive, emotional, and motor functions in patients. The process of aging is a normal physiological process in human life and is accompanied by the aging of the immune system, which is known as immunosenescence. T-cells are an important part of the immune system, and their senescence is the main feature of immunosenescence. The appearance of senescent T-cells has been shown to potentially lead to chronic inflammation and tissue damage, with some studies indicating a direct link between T-cell senescence, inflammation, and neuronal damage. The role of these subsets with different functions in NDs is still under debate. A growing body of evidence suggests that in people with a ND, there is a prevalence of CD4^+^ T-cell subsets exhibiting characteristics that are linked to senescence. This underscores the significance of CD4^+^ T-cells in NDs. In this review, we summarize the classification and function of CD4^+^ T-cell subpopulations, the characteristics of CD4^+^ T-cell senescence, the potential roles of these cells in animal models and human studies of NDs, and therapeutic strategies targeting CD4^+^ T-cell senescence.

## 1. Introduction

The aging of the global population is swiftly progressing, with an estimated 22% of people expected to be over 60 years old by 2050 [1]. Aging is an inherent physiological progression that takes place during the entirety of the human lifespan and is characterized by a gradual decline in cellular and multi-system functions. Although, with the rapid development of healthcare standards, our lifespans are increasing, aging-related diseases continue to trouble people. Therefore, controlling or delaying aging is an important measure facing an aging society.

In recent years, the concept of immunosenescence has attracted researchers’ attention. Immunosenescence refers to the gradual decline and dysregulation of the immune system’s function in the human body. It is typically associated with the aging process and is considered a part of it. This can lead to a reduced ability to effectively eliminate harmful substances such as toxins and pathogens, resulting in a state of chronic inflammation referred to as inflammaging [2]. Consequently, the body’s ability to respond to pathogens and cancer cells weakens over time. With age, adaptive immunity experiences progressive functional impairments and heightened autoimmunity, which can lead to an increased risk of developing NDs [3].

T-cells are the cornerstone of the immune system and have a key function in cellular immunity and humoral immunity through helping B cells. Alterations in CD4^+^ T-cells can trigger persistent inflammation and exacerbate age-related traits across the body, bolstering the viewpoint that T-cell senescence significantly contributes to the broader aging phenomenon. CD4^+^ T-cells are key participants in NDs, capable of causing harmful inflammation [4,5], but are also involved in protecting neurons from inflammatory damage [6,7]. An increasing number of studies have reported the presence of CD4^+^ T-cell subsets with senescent phenotypes in ND patients. This review provides an overview of CD4^+^ T-cell classification and function and the characteristics of CD4^+^ T-cell senescence and summarizes the role of CD4^+^ T-cells’ senescence in NDs. Additionally, we summarize the role of CD4^+^ T-cell senescence in NDs (including MS, AD, PD, and ALS) and elaborate on targeted therapeutic strategies.

## 2. Subsets of CD4^+^ T-Cells and Their Roles in Neurodegenerative Diseases

**Figure 1:**
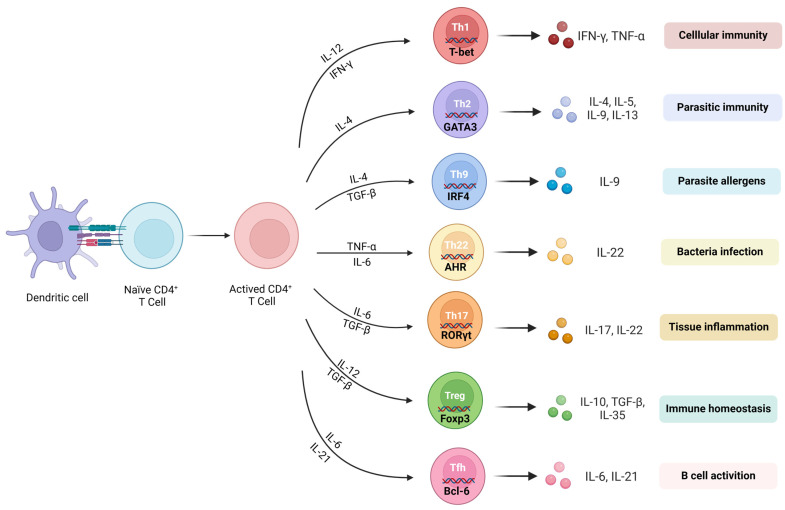
Subsets and functions of CD4^+^ T cells. CD4^+^ TNs initiate immune responses primarily by secreting specific cytokines after activation and differentiation into various effector subtypes. The differentiation of each subset is dependent on the activation of various transcription factors. T-bet, GATA3, IRF4, RORγt, AHR, Foxp3, and Bcl-6 are the transcription factors for Th1, Th2, Th9, Th17, Th22, Tfh, and Treg, respectively. Th1 cells, typically stimulated by intracellular pathogens such as bacteria and viruses, produce IFN-γ and TNF-α. Th1 cells enhance the host’s anti-infection capabilities and promote pathogen clearance by releasing cytokines, particularly IFN-γ, thereby playing a defensive role. Th2 cells secrete cytokines such as IL-4, IL-5, and IL-13 and are involved in mediating immune responses against bacteria, allergens, and toxins. The hallmark cytokine of Th17 is IL-17. Th17 cells can identify and monitor tissue damage and inflammatory conditions in the body and guide the inflammatory response and tissue repair process. Th17 cells interact with and regulate other immune cells and maintain immune system balance through self-regulation. TNs differentiate into Th9 cells under the promotion of TGF-b and IL-4, mainly secreted by Th2 cells. Although Th2 and Th9 share the STAT6 signaling pathway, there are other transcriptional differences that distinguish them. For example, PU.1, an ETS family transcription factor, is highly expressed in Th9 cells and is associated with the secretion of IL-9 by Th9 cells, while inhibiting Th2 differentiation. Treg cells primarily secrete TGF-β and IL-10, demonstrating immunosuppressive functions. Treg cells are mainly involved in immune tolerance and regulation, ensuring an appropriate immune response to foreign antigens and preventing excessive immune responses leading to autoimmune diseases and excessive inflammation, thus maintaining immune system tolerance to self-antigens. Tfh cells are located in B-cell follicles and directly participate in humoral immunity by promoting germinal center (GC) reactions and the differentiation of memory B cells and plasma cells. Tfh cells highly express the master transcription factor Bcl-6, inducible co-stimulatory molecule (ICOS), programmed death-1 (PD-1), and chemokine receptor CXCR5. Tfh-cell differentiation is mediated by the production of IL-6 and IL-21, which induce the expression of the Bcl-6 transcription factor through the STAT3 signal. Initial T cells differentiate into Th22 cells in the presence of IL-6 and TNF-α. The hallmark cytokine of Th22 cells, IL-22, is a member of the IL-10 family, expressing aryl hydrocarbon receptor (AhR) and CCR10.

A CD4^+^ T-cell is a type of adaptive immune cell that originates in the bone marrow and matures in the thymus [8]. Upon activation by antigens, naïve CD4^+^ T-cells (TN cells) can differentiate into different functional subpopulations, including helper T lymphocytes (Th cells), such as Th1, Th2, Th9, Th17, Th22, and follicular helper T-cells (Tfh), as well as regulatory T-cells (Treg cells) and memory CD4^+^ T-cells [9]. Upon activation and differentiation into distinct effector subtypes, CD4^+^ T-cells mediate immune responses by releasing specific cytokines (Figure 1).

### 2.1. Naïve CD4^+^ T-Cells

Naïve T-cells (TNs) are a specific group of T-cells that have left the thymus but have not yet been exposed to any antigens. TNs are considered “uneducated” and are characterized by their capacity to respond to a variety of antigens. TNs normally have the CD45RA, CD62L, and CCR7 phenotype with a lack of CD45RO expression. CCR7 and CD62L are both implicated in T-cell homeostasis in secondary lymphoid organs, with CCR7 playing a significant role in the process of eliminating self-reactive thymocytes, and they also engage with ligands expressed on high endothelial venules (HEVs) [10]. The immature maintenance of T-cells in AD may lead to cognitive impairment, and the age-related downregulation of the immature marker CCR7 on T-cells is associated with cognitive deterioration [11].

### 2.2. Effector CD4^+^ T-Cells

TNs can differentiate into various subgroups of effector CD4^+^ T-cells (Teff) after being activated by antigen-presenting cells (APCs). These Teff-cell subgroups mainly include Th1, Th17, Th2, Th9, Th22, Treg, and Tfh.

The differentiation of Th1 cells relies on the induction of interleukin (IL)-12 and interferon (IFN)-γ. T-bet is the predominant transcription regulator used to regulate the differentiation of Th1 [12]. Th1 secretes cytokines, such as IFN-γ, IL-2, and tumor necrosis factor (TNF)-α, which promote inflammatory responses. Th1 also activates macrophages, produces nitric oxide, and promotes the proliferation of cytotoxic T lymphocytes, leading to the destruction and phagocytosis of microbial pathogens. However, excessive inflammatory responses can lead to unexpected tissue damage.

The differentiation of Th17 cells relies on the synergistic induction of IL-6 and transforming growth factor (TGF)-β. The RORγt plays a crucial role as a transcription factor for Th17 cells. RORγt directly targets the promoters of IL-17A, IL-17F, and IL-22 and induces their expression. Th17 cells primarily secrete IL-17 and are involved in inflammatory responses and autoimmune diseases. The pathogenicity of Th17 cells is influenced by the presence of TGF-β. “Conventional” Th17 cells characterized by the secretion of IL-17 are generated by the combined stimulation of TGF-β and IL-6. In addition, IL-6, IL-1β, and IL-23 induce the production of “pathogenic” Th17 cells characterized by high levels of IFN-γ, granulocyte–macrophage colony-stimulating factor (GM-CSF), and IL-22 [13]. A large number of studies have shown that Th1 and Th17 play important roles in regulating neuroinflammation and subsequent neurodegeneration in many NDs [14,15,16].

Th2′s differentiation is dependent on the induction of IL-4. The regulator for Th2-cell activation, GATA-3, is involved in the differentiation process through signal transduction and transcription activation factors [17]. Th2 cells primarily mediate humoral immune responses, protecting the host against worm infections, promoting tissue repair, and contributing to chronic inflammation such as asthma and allergies. Activated CD4^+^ T-cells can also secrete IL-4, further promoting Th2 differentiation. Additionally, GATA-3 inhibits the development of Th1 cells by suppressing Th1-related genes such as Tbx21, Stat4, and IL-12rb2 [18]. In the peripheral blood of ALS patients, there is a decrease in anti-inflammatory Th2 cells. Additionally, the secretion of IL-10, an anti-inflammatory cytokine, by Th2 cells is significantly reduced in ALS patients. In the peripheral blood of ALS patients, there is a decrease in anti-inflammatory Th2 cells [19].

Th9 cells are a unique population of helper/effector T-cells that promote tissue inflammation [20]. IL-9 is significantly enhanced in AD patients, and it can be either inflammatory or regulatory depending on the background and source of the producing cells [21,22,23], because it affects Th-17-cell differentiation and Treg suppression activity through interactions with STAT3 and STAT5 [24,25].

Th22 is a novel T-cell subset that is distinct from Th17 and other known T-cell subsets, with unique gene expression and function. It is regulated by the aryl hydrocarbon receptor (AHR) and produces cytokines such as IL-22 [26]. Kebir et al.’s preliminary study showed that the upregulation of IL-22R in the brains of MS patients, as well as the cooperative effect of IL-22 and IL-17A, disrupts the integrity of blood–brain barrier (BBB) tight junctions by reducing the expression of occludin in endothelial cells and triggers an autoimmune reaction targeting central nervous system myelin components [27].

Tregs differentiate in response to the induction of TGF-β, and the forkhead box P3 (Foxp3) is their specific transcription factor [28]. Tregs play a crucial role in maintaining immune self-tolerance and regulating the homeostasis of Teff subsets [28,29,30,31]. The control of Foxp3 expression is crucial for the formation, sustainability, and operation of Tregs. When Tregs do not express Foxp3, they exhibit an activated memory T-cell (TM) characteristic and obtain effector abilities such as generating proinflammatory cytokines and prompting the onset of autoimmune disorders [29,32]. The neuroprotective effects of Treg have been proposed in other NDs. Specifically, the number of Treg cells is significantly reduced in patients with ALS, and it is negatively correlated with disease progression [33]. In AD mouse models, the depletion of the Treg population accelerates the occurrence of cognitive deficits, while their amplification determines the increase in the recruitment of protective microglia associated with plaques, thereby improving cognitive function [34].

T follicular helper CD4^+^ T (Tfh) cells are a special subset of CD4^+^ Th cells that have been identified as providing help to B cells in germinal centers (GCs) [35]. Bcl-6 is a major regulatory transcription factor in the differentiation of Tfh cells. T-cells lacking Bcl-6 cannot differentiate into Tfh cells or maintain GC reaction, while the overexpression of Bcl-6 promotes the expression of the Tfh-related molecules CXCR5 and PD-1 [36]. CXCR5 is a prominent surface marker of Tfh cells and is also the most widely used marker for identifying Tfh cells [37]. Tfh-cell differentiation is induced by the production of IL-6 and IL-21, which activate STAT3 signaling to promote the expression of Bcl-6. One study showed that giving IL-21 before inducing EAE enhances inflammation in the central nervous system and worsens the severity of EAE [38].

### 2.3. Memory CD4^+^ T-Cells

Once a pathogen is eliminated, memory T-cells (TMs) persist. Central memory CD4^+^ T-cells (Tcms) represent a pool of less differentiated memory cells and are long-term TMs generated from initial T-cells after antigen stimulation. They can be activated upon antigen restimulation and, with CD4^+^ stem cell-like memory T-cells (Tscms), recirculate in secondary lymphoid organs. Tcm cells have the potential for self-renewal but lack inflammatory and cytotoxic functions [39]. In contrast, effector memory CD4^+^ T-cells (Tems) represent a more differentiated population of circulating effector cells. They can undergo activation and proliferation upon antigen restimulation and perform corresponding immune functions. These cells can rapidly enter tissues and include tissue-resident memory T-cells (Trms), which remain at the initial site of pathogen entry. Tem cells provide a rapid and effective secondary defense response [40]. With age, there is an increase in the number of TMs (Th1 and Th17 of CD4^+^ T-cells) relative to naïve T-cells. Cytokines secreted from these aged T-cells increase the permeability of the BBB, which increases the opportunity for additional immune cells or their products to enter the brain [41,42].

## 3. Features of CD4^+^ T-Cell Senescence

The senescence of CD4^+^ T-cells is primarily characterized by four major hallmarks: thymic involution, imbalance of the TN/TM ratio, loss of plasticity, and metabolic changes. By examining the characteristics of T-cell senescence, we can evaluate the effects of T-cell senescence on human health and explore the feasibility of targeting senescent CD4^+^ T-cells for addressing NDs.

### 3.1. Thymic Involution

Thymic size and cellularity decrease with aging; this is referred to as thymic involution. The thymus plays a fundamental role in the development of T-cells, immune surveillance, and the prevention of immune dysregulation and cancer. Thymic involution initiates during adolescence and, by middle age, the thymus is almost completely replaced by fatty tissue [43,44]. TNs mature in the thymus gland, but as individuals age, the thymus experiences continuous atrophy and thymic output diminishes, resulting in a decline in the amount of peripheral TNs. It is worth noting that while the thymic output of T-cells declines significantly during adolescence, humans can sustain a relatively stable T-cell count for an extended period. This is because the replenishment of T-cells in adults is less reliant on the activity of the thymus and more dependent on the homeostatic proliferation of TNs [45]. Negative selection in the thymus of older adults results in an increase in the production of self-reactive T-cells [46]. In AD patients and elderly individuals, there is an increase in Aβ-specific T-cells [16,47]. These Aβ-specific T-cells can infiltrate the brain tissue and promote AD-like symptoms in mice [16]. Patients with MS present with the signs of early thymic involution and reduced immune functions [48]. The involution of the thymus results in a decline in the production of naive T-cells and reduced T-cell activity [49].

### 3.2. Imbalance of Naïve CD4^+^ T and Memory CD4^+^ T

Thymic degeneration results in the loss of T-cell development and reduced migration of TNs to the periphery, resulting in an increased proportion of Tcms and Tems [50]. T-cells are made to mature in the thymus gland and develop a diverse repertoire of TCRs, which allows them to elicit effective responses to a broad spectrum of antigens, thereby sustaining typical adaptive immune functions. However, as individuals age, the thymus gradually atrophies, causing a reduction in the diversity of the TCR repertoire and rendering older individuals more vulnerable to infections [51].

**Figure 2:**
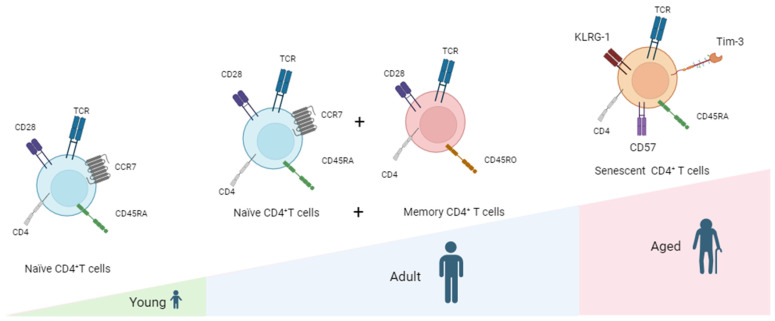
Changes in CD4^+^ T cells during aging. Aging also takes place within the T cells of humans, and as age increases, there are noteworthy modifications in the characteristics and proportions of T-cell subtypes, resulting in an imbalance within the immune system. Age-related thymic atrophy results in decreased adaptive immune function, with the most typical feature being a decrease in TNs and an increase in TMs (including Tcms and Tems). Moreover, senescent T cells notably downregulate the expression of CD28 and exhibit high levels of other aging-related markers such as Tim-3, CD57, and KLRG-1.

With increasing age, T-cells undergo changes in function and quantity, including recognition and response to self-antigens. In addition, the imbalance between TNs and TMs also occurs in NDs. Peripheral T-cells in AD patients and mouse models have been extensively studied, and the reduced ratio of TNs to TMs is a consistent phenotype [52]. In Parkinson’s disease patients, there is a reduced proportion of CD45RA^+^ TNs and an elevated proportion of CD45RO^+^ TMs in their peripheral blood when compared to a healthy control [53]. Furthermore, senescent T-cells exhibit decreased expression of the co-stimulatory signal receptor CD28. There is an observed increase in the prevalence of circulating CD28^−^ T-cells with advancing age, evident in both individuals with autoimmune conditions and those without [54]. In MS brain biopsy samples, CD4^+^CD28^−^ T-cells were detected, which exhibited a cytotoxic phenotype and expressed CX3C chemokine receptor 1 (CX3CR1), a receptor that binds to the chemokine ligand 1 (CX3CL1) with the C-X3-C motif [55]. Due to the upregulation of CX3CL1 in the CSF and brain of MS patients compared to healthy controls, the authors speculate that the recruitment of highly inflammatory T-cells mediated by CX3CL1 occurs in the brains of MS patients. For this reason, these molecules are being used increasingly as biomarkers of senescent T-cells (Figure 2). In addition, several molecules are upregulated in killer cell lectin-like receptor subfamily G member 1 (KLRG-1) and CD57, and T-cell immunoglobulin and mucin-domain containing-3 (TIM-3) are upregulated in senescent T-cells [56].

### 3.3. Decrease in CD4^+^ T-Cell Plasticity

As individuals age, T-cells experience a transition from a steady state of TN to a terminally differentiated Teff stage, which may lead to developmental biases, the loss of plasticity, and reduced immune responsiveness to antigen stimulation. CD4^+^ Th cells play an integral role in mediating many aspects of autoimmune diseases. Exhausted, cytotoxic, and activated T-cells can be identified in the CD4^+^ T-cells of aging mice, often exhibiting proinflammatory characteristics, with the predominant subsets being Th1 and Th17. Both of these subsets share a common feature of producing proinflammatory cytokines, which might contribute to the onset of chronic inflammation, known as inflammaging [57]. Raised levels of circulating inflammatory cytokines are accompanied by the presence of these subsets, primarily including IFN-β, IL-6, and IL-27 [58,59]. This suggests an interaction between inflammation and CD4^+^ T-cell senescence. Peripheral CD4^+^ T-cells from elderly AD patients exhibit biased differentiation, such as increased Th17, Th9, and Th1 activity [60,61,62]. In PD patients, a shift towards Th1 immune response has been reported, including increased IFN-γ production and the reduced number and suppressive capacity of Tregs [63]. In healthy individuals, interconversion between these subsets of CD4^+^ T-cells occurs, aiding responses to various types of antigens. However, in aging individuals, these cells are mostly found in a terminally differentiated stage, losing their ability to transition and their plasticity. This decrease in plasticity could potentially be a factor in the higher risk of age-related diseases.

### 3.4. The Metabolic Changes in CD4^+^ T-Cells

Metabolic changes also occur during CD4^+^ T-cell senescence. The elderly exhibit elevated baseline and maximum oxygen consumption rates, higher ratios of extracellular acidification rates, and increased rates of proton efflux in their CD4^+^ T-cells compared to the young [64]. The PI3K/Akt/mTOR pathway is one of the most important pathways involved in the senescence of resting T-cells. Patients carrying PI3K mutations demonstrate a phenotypic characteristic of senescence in their T-cells, characterized by an elevated abundance of senescent or terminally differentiated T-cells, increased mTOR signaling and glycolysis, and impaired cellular function. This suggests that the mTOR pathway may be a key player in CD4^+^ T-cell senescence, and the use of rapamycin (RAPA) can lower the level of glycolysis by inhibiting the mTOR signaling pathway and promoting the number of naïve T-cells, thereby improving the immune function of the PASLI (p110δ-activating mutation causing senescent T-cells, lymphadenopathy, and immunodeficiency) patients [65]. Studies have shown that prototype inhibitors of glycolysis pathway, 2-deoxyglucose (2-DG) and metformin (MET), reduce the production of IL-17 in Th17 cells and the severity of experimental autoimmune encephalomyelitis (EAE) in mouse models [66,67].

Another study has shown that mitochondrial respiration is damaged in CD4^+^ T-cells of the elderly, which may trigger chronic inflammation [68]. These alterations in metabolism additionally lead to the formation of distinct proinflammatory characteristics in older individuals and might also influence the capability of CD4^+^ T-cells to react effectively to pathogens, contribute to immunosenescence, and shape the overall immune response in aging individuals. Although there is currently a lack of direct comparisons between immune senescence and metabolic changes in neurological diseases, one study found that T-cells lacking mitochondrial transcription factor A (TFAM) and displaying mitochondrial dysfunction act as accelerators of aging, leading to multiple features associated with aging, including neurodegenerative changes [69]. This suggests a potential relationship between immune senescence, immune metabolism, and neurological diseases.

## 4. The Potential Role of CD4^+^ T-Cell Senescence in Neurodegenerative Diseases

Immunosenescence and chronic inflammation are both contributory elements in the pathogenesis of neuroinflammatory conditions. They can lead to an imbalance in anti-inflammatory mechanisms, resulting in persistent, low-level inflammation. Immunosenescence has been reported to be associated with cognitive processes and ND. A phenomenon of dysfunction in the effector functions of senescent CD4^+^ T-cells has recently been observed in patients with NDs (including MS, AD, PD, and ALS).

### 4.1. Multiple Sclerosis

**Figure 3:**
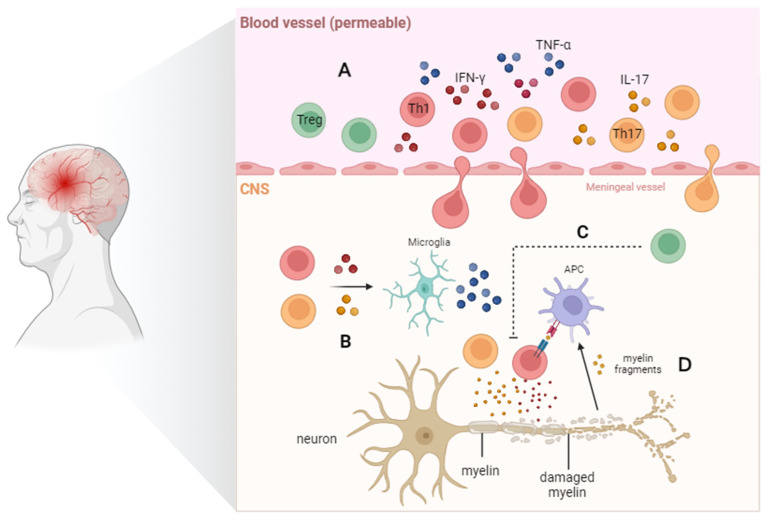
CD4^+^ T cells in elderly patients with multiple sclerosis. T-cell infiltration, particularly of CD4^+^ T helper cells, occurs in the brain tissue of MS patients. (**A**) Th1 and Th17 receive myelin antigens released from the CNS and release proinflammatory cytokines (IFN-γ, TNF-α, and IL-17), leading to increased peripheral inflammation. (**B**) T cells entering the CNS can release proinflammatory cytokines to activate microglial cells. (**C**) The suppressive effect of Treg cells on proinflammatory or autoreactive T cells is impaired in MS. (**D**) Proinflammatory cytokines from Th1 and Th17 cells, as well as TNF-α released by microglial cells, can directly result in demyelination and neuronal damage.

Multiple sclerosis (MS) is a persistent condition that affects the central nervous system (CNS) and is characterized by chronic neurodegeneration, inflammation, and damage to the myelin sheath. With aging, it leads to severe neurological and functional impairments. The immune system plays a role in myelin destruction, ultimately resulting in neurologic dysfunction in patients [70], where immune responses mediated by self-reactive CD4^+^ T-cells play a crucial role in the pathogenesis of MS [71]. The primary shared characteristic of aging and MS is the build-up of CD4^+^CD28^−^ T-cells [72,73]. These T-cells are activated by repeated viral antigen stimulation prior to their migration to the CNS, where they lead to tissue damage and the release of self-antigens. Preliminary studies have revealed an increased presence of CD4^+^CD28^−^ T-cells that preferentially produce IFN-γ in some MS patients [74]. It has been reported that thymic involution is accelerated in patients with relapsing–remitting MS or primary progressive MS [75], and patients with relapsing–remitting MS and secondary progressive MS often experience an early decrease in immune function linked to thymic dysfunction, resulting in reduced levels of TN and compromised function of Treg [48]. Zuroff et al. [76] found a depletion of CD4^+^ TNs and an accumulation of circulating CD4^+^ TMs in patients with MS. Persistent inflammation is typically observed in the brains of elderly patients with MS, confirming the significant accumulation of proinflammatory CD4^+^ T-cells (including Th1 and Th17) in the CNS (Figure 3). Th17 cells possess a large number of chemokines and chemokine receptors required for crossing the blood–brain barrier, allowing them to disrupt the blood–brain barrier and enter the central nervous system through several different pathways. In vitro and in vivo studies have shown that Th17 cells can effectively disrupt tight junctions of the blood–brain barrier through the actions of IL-17A and IL-22, express high levels of the cell-dissolving enzyme granzyme B, and promote additional CD4^+^ lymphocytes to be recruited from the systemic circulation to the central nervous system, which plays an important role in the breakdown of the BBB in EAE [27]. Th1/Th17 cells. These cells can infiltrate the central nervous system early in the course of EAE and may participate in microglial activation, thus playing a crucial role in the development of MS [77].

### 4.2. Alzheimer’s Disease

Alzheimer’s disease (AD) is a type of neurodegenerative disease associated with immunosenescence and characterized by the death of neurons and brain tissue shrinkage. T-cells were detected in the brains of AD patients [78]; these cells are mostly activated and characterized by a memory phenotype [78,79]. Research has found that in AD patients, the T-cell telomere length is significantly shorter than that in healthy controls [80]. Additionally, T-cell telomere length is negatively correlated with AD disease indicators such as elevated plasma TNF-α levels, loss of CD28 expression on T-cells, and increased sensitivity to apoptosis in T-cells [80]. Severe fluctuations in T-cell subsets (a reduction in the proportion of TNs and a rise in the percentage of TMs) have been found in the peripheral blood of Alzheimer’s patients [81]. In comparison to age-matched controls, AD patients exhibit a reduced percentage of CD4^+^ TNs and an increased percentage of terminally differentiated CD4^+^ TMs [82]. Severe fluctuations in T-cell subsets have been found in the peripheral blood of AD patients, with a decreased percentage of TNs, an increased percentage of TMs, and a significant expansion of late-differentiated CD28^−^ T-cells (within the CD4^+^ population) compared to healthy individuals. Recently, reports have shown that healthy individuals with fewer CD4^+^ Tems have better cognitive abilities [83,84]. Elderly AD patients exhibit a biased differentiation in their peripheral CD4^+^ T-cells, including increased Th17 and Th1 activity [61]. Research has shown that the Th17/Th1/Treg ratio is imbalanced in elderly patients with Alzheimer’s disease, with elevated levels of IL-17 and reduced levels of IL-10 detected in their serum and cerebrospinal fluid (CSF) compared to those without the condition; furthermore, an elevated proportion of Tems and decreases in Tregs and TNs have been reported in the sera of these patients [62,85,86,87]. Reports have suggested that IL-17 can induce cognitive and synaptic impairments and compromise the integrity of the blood–brain barrier [88,89]. Conversely, inhibiting IL-17 could mitigate cognitive decline [88,90].

Studies have shown that the accumulation of beta-amyloid protein (Aβ) is related to the occurrence of AD [91,92]. Aβ is produced by the cleavage of the amyloid precursor protein, and it forms aggregates, referred to as “plaques”, which can cause damage to nerve cells and trigger inflammation, leading to cognitive impairment [93,94]. In the process of AD, T-cells can actively damage the BBB and infiltrate into the brain tissue. T-cells can cause BBB damage by reducing endothelial integrity and stimulating astrocytes [14,42]. There seems to be a vicious cycle between the accumulation of Aβ and the injury of endothelial cells and astrocytes. The proinflammatory cytokines produced by reactive CD4^+^ T-cells further exacerbate this cycle [16]. McManus and his colleagues have demonstrated in their study on the impact of infection on AD pathology that not only Th1 cells but also Th17 cells infiltrate the brain from the bloodstream during the progression of AD [42]. Machchi et al. [15] found in experimental studies on AD mice (APP/PS1) that CD4^+^ T-cells can accelerate the progression of AD, mainly confirming that the subtypes Aβ-Th1 and Aβ-Th17 of effector T-cells can promote the occurrence of pathological changes in AD through downregulating the action of Treg cells in the periphery nervous system and CNS, while simultaneously increasing the activation of microglia and neural inflammation, forcing memory impairment to worsen, and the transfer of Aβ-reactive Th1 and Th17 into mice showing accelerated behavior and pathology, thus supporting their roles as disease instigators. Early experiments conducted by Browne et al. [16] confirmed that infiltrating T-cells in the brain can also produce IFN-γ, promoting increased activation of microglia, Aβ deposition, and cognitive impairment. In an Aβ_1–42_-induced AD rat model, Th17 cells infiltrated the brain parenchyma and produced IL-17 and IL-22 [14]. In one study, it was found that IL-17 significantly increased in the hippocampus, peripheral blood, and cerebrospinal fluid of AD rats induced via injection with Aβ peptide [14,95]. This indicates that Th17 cells play a significant role in the progression of AD. Research by Zenaro et al. [96] also showed that Aβ aggregates can mediate the recruitment and chemotaxis of neutrophils to produce IL-17, which is directly toxic to neurons and the BBB and may amplify neutrophils in the central nervous system, leading to pathological deterioration. Furthermore, a clinical study indicated that Aβ-reactive T-cells were more readily detectable in the peripheral blood of AD patients and healthy elderly subjects compared to a middle-aged control group [47]. In AD patients, it has been found that the progression of cognitive impairment is related to Th17 cells and c-Jun N-terminal kinase (JNK) pathway-associated phosphatase (JKAP), which plays a key role in regulating inflammation and immune responses. JKAP and Th17 cells are dysregulated and inter-related in AD [97]. An acidic α-glucosidase (GAA) isolated from ginseng has been found to alleviate neuroinflammation in AD mice by regulating the imbalance of the Th17/Tregs axis [98]. The above studies suggest that restoring the function of peripheral senescent CD4^+^ T-cells may be a potential strategy for improving the condition of AD.

### 4.3. Parkinson’s Disease

Parkinson’s disease (PD) is a neurodegenerative disorder of the central nervous system that primarily affects movement. It is characterized by the death of dopaminergic cells in the substantia nigra region of the brain, which occurs more frequently with age. Its characteristic is the loss of dopaminergic neurons (DN) in the substantia nigra (SN). T-cells play a critical role in the pathogenesis of PD [63,99]. The main pathological manifestations of the disease include neuron apoptosis and the deposition of α-synuclein in the substantia nigra [100]. In recent years, many studies have shown that α-synuclein is generally distributed in the brains of Parkinson’s disease patients [101]. Researchers propose that abnormal misfolding of α-synuclein causes neuroinflammation and lysosomal membrane permeability, leading to calcium influx and ion homeostasis disruption, resulting in neuronal toxicity and DA neuron apoptosis [102,103]. Research findings in MPTP-mediated PD mouse models suggest that α-synuclein promotes the polarization of CD4^+^ T-cells towards Th1 and Th17 phenotypes, as well as the dysfunction of Treg [104], leading to neuronal apoptosis [63]. It has been found that CD4^+^ T-cells infiltrate the brain in PD mouse models and are the main mediators of dopamine toxicity [63]. In PD mouse models, the key role of CD4^+^ T-cells in enhancing microglial activation and promoting neurodegeneration supports the idea that CD4^+^ T-cells play a critical role in enhancing microglial activation and promoting neurodegeneration [105]. PD patients also experience the disruption of the BBB, allowing peripheral CD4^+^ T-cells to infiltrate the brain and potentially affect other mechanisms related to neurodegeneration, such as oxidative stress and mitochondrial dysfunction [106]. Some evidence obtained from PD mouse models supports the notion that T-cell-mediated dopaminergic toxicity plays a significant role in PD, which is almost entirely mediated by CD4^+^ T-cells producing IFN-γ [63]. The removal of CD4^+^ T-cells greatly reduces MPTP-induced dopamine neuron death in PD mice. Additionally, transferring purified Treg cell groups weakens MPTP-mediated neuroinflammation and neurodegenerative activity in PD mice. In PD mouse models lacking IL-17A, symptoms such as motor impairment, dopaminergic neuronal degeneration, and BBB disruption are alleviated [63].

A study conducted by Fiszer [107] has shown that PD patients exhibit a reduced proportion of CD45RA^+^ TNs and an elevated proportion of CD45RO^+^ TMs in their peripheral blood when compared to healthy individuals. In addition, studies by He et al. [53] have found that compared to healthy controls (HCs) unaffected by Parkinson’s disease (PD) medications, PD patients show an increase in quantities of late-differentiated CD4^+^ T-cells (CD3^+^CD4^+^CD28^−^CD27^−^). Studies have also suggested that people with PD tend to develop a Th1 immune response characterized by heightened levels of IFN-γ and reduced quantities and suppressive capabilities of Tregs [108,109,110,111]. Research has revealed that Th17 levels are elevated in the circulation and post mortem brain tissue of PD patients, and the IL-17 produced by Th17 cells binds to IL-17R expressed on midbrain neurons, inducing neuronal death through the upregulation of nuclear transcription factor-κB and downstream signaling pathways [112]. Analysis of the brain in PD has revealed elevated quantities of microglia and immune cells entering the CNS and peripheral cells, as well as heightened levels of proinflammatory cytokines, including IL-6, TNF-α, IL-1β, and IFN-γ [113,114].

### 4.4. Amyotrophic Lateral Sclerosis

Amyotrophic lateral sclerosis (ALS) is a disease impacting nerve cells in the brain and spinal cord, causing gradual degeneration and the eventual demise of motor neurons, with an increasing incidence rate with age, peaking between 60 and 79 years old [115]. ALS patients gradually lose control of their muscles, eventually leading to severe muscle atrophy and paralysis. ALS typically affects movement, speech, and swallowing functions [116]. Increasing evidence indicates that neuroinflammation plays a crucial role in the progression of ALS [117]. Aging leads to aberrant activation of microglia and excessive secretion of proinflammatory cytokines, exacerbating neuroinflammation and neurodegeneration in ALS patients. Studies have indicated changes in the distribution and activation status of T-cells in individuals with ALS compared to healthy individuals. The systemic elevation of aging and late TMs is characteristic of rapidly progressing ALS and bulbar-onset ALS [118]. In ALS patients, there is an increased proportion of Th1 cells and a decreased proportion of anti-inflammatory Th2 [19]. Additionally, in comparison to healthy controls, ALS patients show a significantly elevated percentage of Th17 and a significantly decreased percentage of Treg [119,120,121]. Furthermore, ALS patients have significantly lower serum levels of IL-10 and IL-4 in comparison to healthy controls, while IFN-γ and IL-17 levels are significantly higher. This indicates that the suppressive function of Th2 and Treg on immune response is compromised in ALS patients [122], which may be the basis for the expansion of Th1 and Th17. Other clinical evidence shows that ALS patients have significantly decreased numbers of Tregs, which cannot effectively suppress immune responses [123,124]. Treg cells play a key role in maintaining immune self-tolerance and immune homeostasis [125]. They may migrate to the site of injury in the central nervous system and alleviate neuroinflammation by interacting with local microglia and/or secreting anti-inflammatory cytokines and neurotrophic factors [126]. However, current research only shows proinflammatory shifts in the peripheral immune system in ALS, and further research is needed to understand the roles of Treg/Th2 and anti-inflammatory cytokines (such as IL-10) in the development of ALS.

Transactive response DNA-binding protein 43 (TDP-43) is a transcription activator that mainly participates in RNA splicing and stability and is considered to be one of the factors playing an important role in NDs such as ALS. The abnormal aggregation of TDP-43 has been observed in the brains and spinal cords of ALS patients [127]. Researchers believe that abnormal TDP-43 may interfere with normal neural signal transmission and biological processes necessary for cell survival, leading to neuronal death and disease progression [128]. CD4^+^ T-cells can recognize proteins related to NDs, indicating the autoimmune characteristics of NDs [129,130,131]. As mentioned above, Aβ can stimulate the production of IL-17 in Th17 cells and promote their inflammatory response, accelerating the development of AD. Furthermore, α-synuclein peptides in PD mouse models can be recognized by CD4^+^ T-cells and promote the differentiation of T-cells towards Th1 and Th17. Therefore, we speculate that the aggregates of TDP-43 in ALS patients may be recognized by CD4^+^ T-cells and trigger an autoimmune response in the CNS.

**Figure 4:**
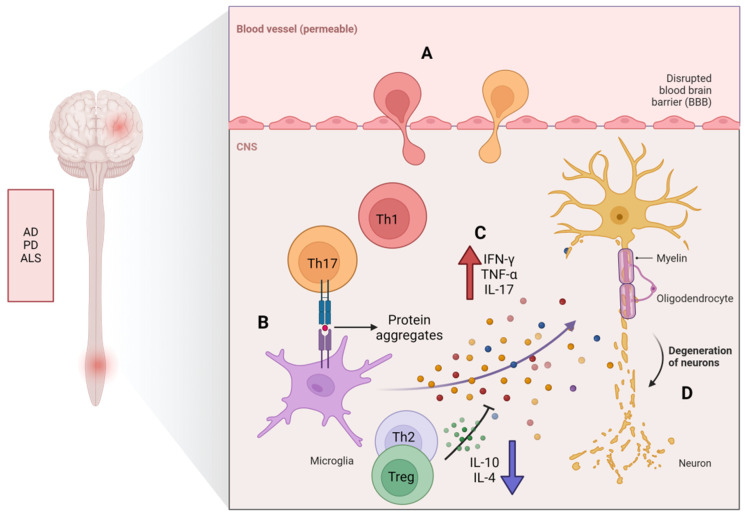
Senescent CD4^+^ T cells and neurodegenerative diseases (AD, PD, and ALS). With age, there is a decrease in the population of TNs, while TMs, notably Th1 and Th17, experience progressive accumulation. (**A**) Senescent T cells promulgate the secretion of cytokines that augment the permeability of the BBB, facilitating their translocation into the central nervous system (CNS). (**B**) Aggregates of misfolded proteins (e.g., Aβ, α-synuclein or TDP-43) are exposed to post-translational modifications, culminating in the genesis of neoepitopes derived from protein aggregates that are perceived as non-self-antigens by the microglial cells within the CNS. (**C**) The liberation of proinflammatory cytokines upon exposure to antigen-presenting T cells precipitates an autoimmune reaction that instigates neurodegenerative changes, ultimately manifesting in neuronal loss. (**D**) Cumulatively, the proinflammatory cytokines that are secreted by both T cells and microglial cells foster neuronal degeneration and subsequent neurologic decline.

The SOD1 gene encodes the enzyme superoxide dismutase 1. This gene provides the instructions for making the superoxide dismutase enzyme (SOD), which is responsible for clearing superoxide free radicals within cells to protect them from oxidative damage. Mutations of the SOD 1 gene are an important factor in familial ALS cases, accounting for about 20% of these cases, and they are the reason for about 2% of sporadic ALS cases [132]. Because of the swift advancement of symptoms, SOD1 transgenic mouse models have been extensively employed in ALS research [133]. Various mutant SOD1 transgenic mice exhibit analogous disease initiation mechanisms such as neuroinflammation, paralysis, and premature death [132]. Compared to those from an early-onset SOD1G93A mouse model, splenocytes from a late-onset SOD1G37R mouse model showed a significant rise in the effector memory CD4^+^ T-cell ratio and a reduction in the TN population ratio [134] (Figure 4).

## 5. The Dawn of Targeting Senescent CD4^+^ T-Cells for the Treatment of Neurodegenerative Diseases

Targeting senescent CD4^+^ T-cells has become an appealing approach for potential therapeutic interventions to recover the function of the nervous system and offer new immune-modulating treatments for NDs.

### 5.1. Adoptive Transfer of Treg

Tregs can oversee the progression of autoimmune diseases. The rapid onset of cognitive decline is related to the early decline of Tregs [135]. Tregs are believed to suppress excessive inflammation and protect neurons, which could positively influence cognition [136]. Replenishing Tregs can enhance cognitive function.

Multiple clinical trials are currently focused on PD and ALS with the objective of utilizing medications to elevate the proportion of endogenous Tregs or expand Tregs outside the body and reintroduce them into patients. The initial findings indicate that the autologous transfer and expansion of Tregs can enhance both the quantity and suppressive capabilities of Tregs and potentially slow ALS progression [137]. The first phase I human study of autologous transplantation of expanded Tregs in ALS patients has been completed, not only proving safety but also resulting in increased Treg numbers and enhanced Treg inhibitory function, slowing down the disease progression speed in ALS patients, supporting the utility of Treg inhibitory function as a meaningful indicator of clinical status [137,138]. Additionally, further understanding of the process of brain APC infiltration and synaptic formation can be utilized to develop new immune modulation approaches, such as using antigen-specific Tregs or chimeric antigen receptor (CAR) T-cells targeting central nervous system-specific antigens [139].

### 5.2. Monoclonal Antibodies

**Figure 5:**
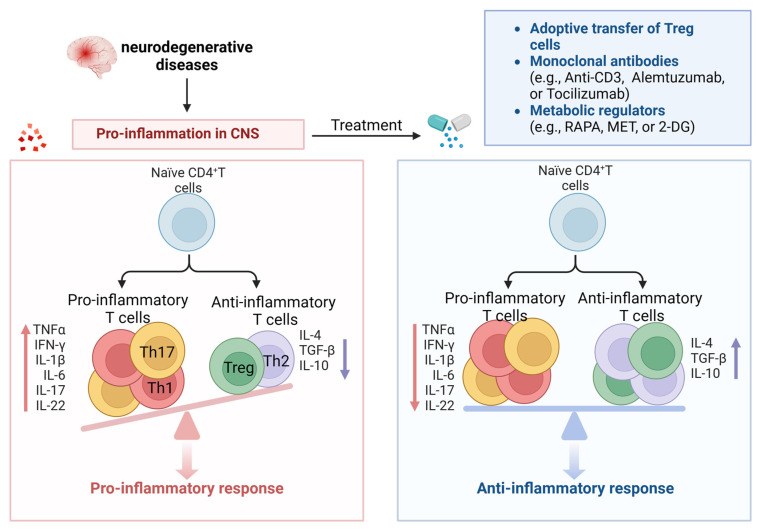
Potential treatment options for neurodegenerative diseases. T-cell subsets in patients with NDs exhibit abnormal activation, such as the overactivation of Th1 and Th17 subsets, leading to harmful inflammation in the nervous system. In addition, the impaired function of Th2 and Treg is an important factor in the occurrence of NDs. The utilization of the adoptive transfer of Treg, monoclonal antibodies (e.g., anti-CD3, alemtuzumab, or tocilizumab), or metabolic regulators (e.g., RAPA, MET, or 2-DG) can effectively foster the differentiation of Treg and, concurrently, impede the differentiation of proinflammatory T cells. Therefore, the therapeutic targeting of the balance between Th1/Th17 and Th2/Treg has become a very interesting research direction that may help to develop new treatments or intervention measures to treat NDs.

It has been discovered that anti-CD3 monoclonal antibodies provide immune tolerance by inducing Treg proliferation and function. Anti-CD3 monoclonal antibodies induce apoptosis or anergy in activated T-cells through several mechanisms. When these antibodies target the CD3/TCR complex on activated T-cells, it disrupts the signaling pathways required for T-cell activation and triggers apoptosis or anergy in activated T-cells while preserving Tregs [140,141]. Alemtuzumab is a humanized IgG1 monoclonal antibody, and recent clinical studies on relapsing–remitting multiple sclerosis patients have shown that alemtuzumab increases anti-inflammatory cytokines (such as IL-10 and TGF-β) during the first six months of treatment, reduces proinflammatory cytokines (such as IFN-γ, IL-17, IL-6, and TNF-α), and increases the percentage and function of Tregs after 24 months of treatment [142]. Tocilizumab is a humanized IgG1 monoclonal antibody against the IL-6 receptor [143], and IL-6 has been shown to induce proinflammatory Th17 cells in MS patients [144]. Furthermore, elevated expression of IL-6 in the brains of PD patients has been reported [106], and Th17 cells secrete proinflammatory cytokines and other detrimental inflammatory factors such as TNF-α, IL-1β, and IL-6, which are associated with the activation of these factors. Th17 cells and inflammatory factors promote inflammatory reactions and neuronal apoptosis. This suggests that tocilizumab may inhibit the proinflammatory function of Th17 cells, making it an attractive candidate drug for ND treatment (Figure 5).

Therefore, manipulating the proinflammatory environment in ND through the monoclonal-antibody-mediated neutralization of inflammatory cytokines may serve as an innovative therapeutic approach to restore the regulatory function of Tregs and promote their proliferation, thereby augmenting tolerance mechanisms.

### 5.3. Hormone and Cytokines

Thymic involution is one of the main features of T-cell senescence, which results in a decrease in the generation of TNs. Therefore, inhibiting thymic involution can serve as a new method to delay T-cell senescence and treat NDs. Decreases in the production of growth hormone (GH), insulin-like growth factor 1 (IGF-1), and keratinocyte growth factor (KGF) can accelerate thymic involution. The administration of growth hormone-releasing peptides leads to a considerable augmentation in thymus size, cell numbers, and T-cell output in aged mice, resulting in a significant increase compared to untreated mice of the same age. Growth hormone treatment can promote the regeneration of thymic epithelial cells and initiate thymic rejuvenation in comparison to age-matched untreated mice [145], and the aging thymus starts to undergo restructuring and compartmentalization, transforming into an ideal organ for thymocyte growth and development. This enhances the production of naïve T-cells with a broader TCR repertoire. The intraperitoneal injection of GH3 (prolactinoma cells capable of producing GH) in aged rats can reverse age-dependent thymic involution and increase the number of thymic cells [146]. Similar results were obtained with the injection of IGF-1 [147]. The use of KGF as a means to promote thymic regeneration has been explored in vitro. Early studies by Min et al. in this field showed that KGF treatment can improve the function of TEC and enhance thymic function in aged mice [148]. KGF treatment in aged mice restored clear cortical and medullary zones and increased the output of naïve CD4^+^ T-cells.

In addition, targeted drugs for hormones can alleviate the symptoms of NDs by restoring thymic function. Leuprolide is a luteinizing hormone-releasing hormone (LHRH) agonist that inhibits the production of sex steroids, restores T-cell immunity by increasing thymic activity [149], and has been found to improve the neuropathological processes in APP/PS1 mice and delay cognitive loss in a subgroup of AD patients [150,151].

Treatment with the cytokine IL-7 can induce the maturation and differentiation of thymic cells into TNs, enhancing the diversity of the TCR [152]. Schluns et al. demonstrated that IL-7 is necessary for the steady-state expansion of immature CD4^+^ T-cells, and IL-7 treatment restored thymic generation in IL-7-deficient mice [153]. Additionally, IL-22 may contribute to thymic regeneration in mice. After the exhaustion of CD4^+^CD8^+^ double-positive thymocytes, IL-22 could potentially boost the thymic regeneration process [154]. The actions of these cytokines help to enhance thymic function and facilitate T-cell generation and differentiation.

### 5.4. Metabolic Regulators

Due to changes in metabolic activity during T-cell senescence, many studies have attempted to manipulate metabolism to maintain T-cell function and inhibit senescence [155]. The mTOR signaling pathway is an important cellular signaling pathway involved in regulating cell growth, metabolism, and survival. mTOR plays a fundamental part in the formation and operation of diverse immune cells, which are integral to the pathology of MS. In a pro-aging environment, mTOR is abnormally activated, and inhibiting mTOR can improve cellular function, improve immune response, and extend lifespan [156]. In vitro and in vivo data suggest that dysregulated mTOR pathways participate in the pathogenesis of MS. In both MS patients and EAE mouse models, TNs are prone to differentiating into Th17, which is associated with abnormal activation of mTORC1 [157]. Glycolysis promotes the differentiation of Th1 but inhibits the differentiation of Treg [158,159]. A study by Gu et al. found that miR-99a downregulated glycolysis mediated by mTOR in the CD4^+^ T-cells of EAE mice, regulating the differentiation of Th1 and Treg and alleviating the progression of EAE [160]. In senescent T-cells, the mTORC1 signaling pathway is upregulated [161]. Studies have been conducted on mTOR inhibitors (such as RAPA and everolimus), which have emerged as prospective treatments for various age-related diseases, particularly in all four primary animal models of aging: worms, flies, yeast, and mice [162,163]. Positive effects reported for mTOR inhibitors include prolonging lifespan and reducing immunosenescence. Based on this, mTOR inhibitors may be potential candidate drugs for targeting senescent CD4^+^ T-cells in ND.

Metformin (MET), a glycolytic pathway inhibitor, has a history of over 60 years due to its safety and has been used as a first-line treatment drug for Type 2 diabetes. By activating AMPK to reduce the glycolytic rate [155,164], it can promote the survival of functional memory T-cells and prevent the accumulation of terminally differentiated senescent T-cells. In an EAE mouse model, MET enhanced Treg differentiation and impaired Th1/Th17 differentiation by inhibiting glycolysis in CD4^+^ T-cells, and MET can alleviate EAE symptoms by reducing Th17 cells and inflammatory cytokines [66]. It has been reported that MET targets systems related to aging through multiple modes of action, including inhibiting mTOR signaling, reducing ROS, and inhibiting complex I in the electron transport chain (ETC) [165]. According to a clinical study conducted by Yang et al., MET can increase the concentration of telomerase and the frequency of undifferentiated T-cells, which may mediate its anti-aging effects [166]. In addition, 2-DG is an inhibitor that blocks hexokinase (the first rate-limiting enzyme in glycolysis) to inhibit the glycolytic pathway [167]. Lewis et al. used the Th17-polarized transfer model of EAE, where they transferred DLNs of MOG peptide-cultured EAE mice treated with 2-DG to immune-deficient mice [67]. They found that the 2-DG-treated cells showed significantly reduced abilities to induce EAE, with decreased leukocyte infiltration and spinal cord inflammation. Therefore, blocking glycolysis alters Th17- and Treg-cell differentiation and protects mice from EAE.

In summary, these interventions targeting metabolism can regulate T-cell metabolism and senescence, showing promising therapeutic results (Figure 5).

## 6. Conclusions

Aging, as a common biological phenomenon, often accompanies a decline in organ function. Due to the immune system’s normal function of clearing aging cells, some researchers have indicated that immunosenescence is possibly an important factor that may contribute to the aging of various organs in the body. Simultaneously, aging can result in impaired immune system function. T-cells are inevitably affected by aging. This includes thymic atrophy, the reduced plasticity of CD4^+^ T-cells, metabolic abnormalities, and various alternations in the proportions of T-cell subsets. CD4^+^ T-cell senescence observed in ND patients and animal disease models contributes to the onset and progression of the diseases. As mentioned above, similar characteristics of CD4^+^ T-cell senescence are observed in different NDs, mainly manifested as decreased TNs, increased TMs [168], the significant enhancement of terminal differentiated CD4^+^CD28^−^ T-cells [53,86,169], and chronic neuroinflammation caused by the infiltration of proinflammatory CD4^+^ T-cells (Th1 and Th17) [62,170]. Currently, some research on CD4^+^ T-cells in ND patients and mouse models has found that inhibiting or removing inflammatory CD4^+^ T-cells (such as Th1 and Th17) or promoting the differentiation of anti-inflammatory T-cells (such as Treg) can alleviate the symptoms of NDs [63,98,171].

Although a large body of research proves a strong association between CD4^+^ T-cell senescence and NDs, many questions remain unanswered. The reasons for CD4^+^ T-cell senescence in neurological disorders and the specific mechanisms through which senescent CD4^+^ T-cells promote neuroinflammation are not fully understood. According to the conventional view, aging results in the decreased function of T-cells or T-cell senescence. However, there is still a lack of relevant research on the treatment of NDs by targeting T-cell senescence. More studies are required to confirm this possibility.

## 7. Future Perspectives

A significant amount of research has been undertaken to investigate different interventions during the process of CD4^+^ T-cell senescence for the prevention and treatment of ND. However, most of these studies are still in the experimental stage. When considering cell-based therapy, factors such as cell storage conditions, economic considerations, the ability to maintain immune cell viability during long-term storage, the quantity and effectiveness of cell engraftment, and post-transplant safety without excessive immune reactions should be taken into account. The safety and potential side effects of targeted drug interventions are also unknown. It is uncertain whether simply supplementing young immune cells or pharmacologically intervening in aging immune cells can effectively maintain their function in the long term. In conclusion, understanding the mechanisms underlying CD4^+^ T-cell senescence can help researchers to design more efficient prevention and therapy strategies for age-related ND, ultimately improving the quality of life of the elderly.

## Data Availability

Not applicable.
